# The Role of General Attitudes and Perceptions Towards Vaccination on the Newly-Developed Vaccine: Results From a Survey on COVID-19 Vaccine Acceptance in China

**DOI:** 10.3389/fpsyg.2022.841189

**Published:** 2022-05-31

**Authors:** Rize Jing, Hai Fang, Hufeng Wang, Jiahao Wang

**Affiliations:** ^1^School of Public Administration and Policy, Renmin University of China, Beijing, China; ^2^School of Public Health, Peking University, Beijing, China; ^3^China Center for Health Development Studies, Peking University, Beijing, China; ^4^Chinese Center for Disease Control and Prevention Joint Center for Vaccine Economics, Peking University Health Science Center, Beijing, China

**Keywords:** vaccination, vaccine hesitancy, perceptions, COVID-19, newly-developed vaccine, China

## Abstract

**Background:**

Vaccination has been considered one of the most effective public health interventions. In the context of the global epidemic of coronavirus disease 2019 (COVID-19), it remains unclear what role general vaccination attitudes and perceptions have on the acceptance of COVID-19 vaccine.

**Objective:**

This study aims to explore the impact of general attitudes and perceptions toward vaccination on the acceptance of a newly developed vaccine, taking COVID-19 vaccines as an example.

**Method:**

A cross-sectional survey was conducted among 2,013 Chinese adult participants. Generalized order logistic regression and path analysis models were used to analyze impacts of general attitudes and perceptions toward vaccination on the acceptance of the COVID-19 vaccine.

**Results:**

The prevalence of hesitancy to vaccination in general is 49.9% among the Chinese adult population. General perceptions of vaccination were associated with corresponding perceptions of the COVID-19 vaccine. A “no hesitancy” attitude toward vaccination is a significant determinant (aOR = 1.77, 95% CI = 1.36–2.31) of future COVID-19 vaccination compared to vaccine refusers, and perceptions of COVID-19 vaccine remain a significant determinant for the acceptance of the COVID-19 vaccine. Path analysis indicates that perceptions of the importance and safety of vaccination have a positive overall effect on the acceptance of the COVID-19 vaccine, and that general perceptions of vaccination as a whole on each measure indirectly influence the acceptance of the COVID-19 vaccine.

**Conclusion:**

General attitudes and perceptions toward vaccination were associated with those of the COVID-19 vaccine and future vaccination intention. To prepare for possible emergence of diseases in the future, routine health campaigns should be launched by relevant government departments and vaccination authorities to enhance the overall awareness and knowledge of vaccination among the public and to ensure optimal vaccination experience. In addition, targeted knowledge dissemination and mass mobilizations should be urged for newly developed vaccines when some specific infectious diseases emerge, such as COVID-19 at present.

## Introduction

Vaccination has been considered one of the most effective public health interventions, especially in preventing infectious diseases (World Health Organization, 2019). For example, following the successful eradication of smallpox and poliomyelitis, the global expansion of children immunization programs has been contributing to substantial reductions in the burden of vaccine-preventable diseases each year (Greenwood, 2014; World Health Organization, 2019). Moreover, with growing recognition of the potential of vaccination in improving population health and reaping macro socioeconomic benefits, new technologies are being invented, and novel vaccines have been developed, tested, and put into practice against a wider spectrum of diseases, including dengue, HIV, and Ebola, for a wider range of populations such as young adults and senior citizens (Rappuoli et al., 2011; Jit et al., 2015; Maruggi et al., 2019; Excler et al., 2021).

However, in terms of demand, negative public attitudes and perceptions toward vaccination, or vaccine hesitancy, have proliferated in recent decades, hindering the success of ongoing vaccination programs and becoming one of the major threats to global health (World Health Organization, 2014; Dubé and MacDonald, 2016; de Figueiredo et al., 2020). Vaccine hesitancy, characterized by delays in accepting or refusing vaccinations despite the availability of vaccination services, has been highlighted as a complex phenomenon with various determinates such as demographic, locational, temporal, and other contextual factors (World Health Organization, 2014). An issue to note, however, is that delays should be assessed within a specific time period. In recent years, there has been growing evidence of vaccine hesitancy due to psychological and social-cognitive factors, for example, lack of trust in the importance, safety, or effectiveness of vaccines, as reported by the World Health Organization (WHO) Strategic Advisory Groups of Experts (SAGE) working group and other researchers (Wheelock et al., 2013; Brewer et al., 2017; de Figueiredo et al., 2020; Dyda et al., 2020; Nowak et al., 2020; Barello et al., 2021; Murphy et al., 2021). The vaccination history of one specific type of vaccine has been reported as a determinant of vaccine acceptance on the same vaccine, particularly in studies regarding influenza vaccines on perceptions of the public or caregivers toward expended immunization programs for young children (Marlow et al., 2007; Shahrabani and Benzion, 2012; Wheelock et al., 2013; Mendel-Van Alstyne et al., 2018; Valido et al., 2018; Dyda et al., 2020).

Ten years after the previous influenza A (H1N1) pandemic, the current coronavirus disease 2019 (COVID-19) pandemic has caused a severe disease burden globally, and this has made vaccination, especially with newly developed vaccines, once again a pressing public concern and a global focus (Huang et al., 2020; Lurie et al., 2020). As the primary goal of vaccination programs is to encourage coverage as soon as possible to establish herd immunity, numerous studies have been conducted around the world to investigate the willingness of the public to accept potential COVID-19 vaccines and to identify potential vaccine hesitancy (Lin C. et al., 2020; Neumann-Bohme et al., 2020; Wang et al., 2020, 2021a; Allen et al., 2021; Lazarus et al., 2021; Murphy et al., 2021; Robinson et al., 2021). However, from a broader perspective, previous findings on vaccine hesitancy may still limit the means of preparing for possible future infectious diseases with newly developed vaccines, but also provide case-by-case information on specific types of vaccine (e.g., influenza). Therefore, their findings would help explain future perceptions of the same vaccine or predict future vaccination behavior rather than for any other new vaccines targeted for new diseases. Although the vaccination history of a particular type of vaccine (e.g., influenza) has been reported as a determinant of acceptance for the same vaccine, only few studies have examined whether the general attitude and perceptions toward vaccination influence the perceptions of a newly developed vaccine, as well as its extent and possible mechanism (Marlow et al., 2007; Shahrabani and Benzion, 2012; Wheelock et al., 2013). An Italian study indicated that skepticism about the safety, efficacy and importance of vaccines was associated with hesitancy to vaccinate their children among pregnant women (Rosso et al., 2019), but the study did not directly point to the association between perceptions of vaccination as a whole and acceptance of a newly developed vaccine. A study from France reported that baseline vaccine hesitancy level in general (not specific to COVID-19 vaccine) was associated with intention to get vaccinated against COVID-19, but no information was available on perceptions of vaccination itself (Detoc et al., 2020). Little evidence exists to report baseline attitudes and perceptions of the general population toward newly developed vaccines. Recently, with the development of multidiscipline perspectives, especially psychology, and analytical approaches, studies have been conducted to examine related issues, for instance, a study that evaluated attitude traits toward vaccination and correlation with vaccination compliance in Israel, and a study that identified psychological characteristics associated with COVID-19 vaccine hesitancy and resistance in Ireland and the United Kingdom (Velan et al., 2012; Ernsting et al., 2013; Williams, 2016; Brewer et al., 2017; Low et al., 2017; Scherr et al., 2017; Murphy et al., 2021).

With the development and approval of domestically produced COVID-19 vaccines, including Sinopharm, Sinovac, and CanSino, China launched a national vaccination program on 31 December 2020. Furthermore, since late September 2021, Chinese residents who have received at least two doses of COVID-19 vaccine 6 months ago have been receiving a booster dose (Lai et al., 2021). To date, China has made significant progress in promoting COVID-19 vaccination. As for February 2022, more than 3.12 billion doses of COVID-19 vaccines have been administered in China, including 554.7 million booster doses (Statista, 2022a,b). A longitudinal study among Chinese adults after 6 months of national vaccination campaign has shown that having a prior vaccination intention was a significant predictor of vaccine uptake (Wang et al., 2022). In China, which has a large population base, the coverage of non-expanded program of immunization (EPI) vaccines, especially that of vaccines for adults, is at a low level worldwide (Hu et al., 2014; Wang et al., 2018; Zheng et al., 2018). The coverage rate of influenza vaccine in the general population was approximately 9.4%, and vaccination rate just reached 37.3% even in the previous pandemic influenza (Wang et al., 2018; Zheng et al., 2018). It has been reported that the increase in vaccine-related incidents in recent years, such as the illegal sale of vaccines in 2016 and the Changchun vaccine incident in 2018, has reduced public confidence in vaccines (Cao et al., 2018; Du et al., 2020). Therefore, before the launch of COVID-19 vaccine campaigns and during a period of good containment of the COVID-19 pandemic in China, this study first assessed vaccine hesitancy level and perceptions of vaccination as a whole among the adult population, and then specifically assessed those of future COIVD-19 vaccines. The study intends to develop an understanding of the prevalence of vaccine hesitancy in general, and to examine the impact and mechanisms of the general attitude and perception toward vaccination on those of future newly developed vaccines. This evidence would help inform preparations and strategies for the prevention and control of the current COVID-19 pandemic and future emerging infectious diseases.

## Materials and Methods

### Study Design, Population, and Sampling

An anonymous online cross-sectional survey was conducted between November and December 2020, approximately 1 month before the COVID-19 vaccine became available to the public, and a vaccination campaign was launched in China. The survey was part of a research project conducted among the adult population in China, performing consecutive investigations to evaluate public vaccine hesitancy, acceptance, and perception of a future COVID-19 vaccine in different stages of the COVID-19 pandemic (Wang et al., 2020, 2021a). Study design, target population, sampling method, and sample source have been reported in detail in previous studies (Wang et al., 2020, 2021a). The survey was conducted on the largest online survey platform, Wen Juan Xing (Changsha Ranxing Information Technology Co., Ltd., Hunan, China). The Wen Juan Xing sample database comprises over 2.6 million respondents, whose personal information was confirmed, allowing for an authentic, diverse, and representative sample. The target population was Chinese adults aged 18 years or above residing in mainland China, and a stratified sampling method by age and location was adopted to match adult respondents in the Wen Juan Xing sample database (Wang et al., 2020, 2021a). This survey included 791 participants who had been successfully followed up since the first survey in March 2020 and 1,222 newly recruited participants, making a total sample size of 2,013. The study was approved by the Peking University Institutional Review Board (IRB00001052-20,011). Informed consent was given by each participant upon the completion of the questionnaire.

### Measures

The self-administered questionnaire consisted of three parts. The first part included questions on the situation of the pandemic and basic demographic characteristics of the participants and such as age, gender, education, marital status, employment status, total household income in 2019, health status, chronic disease status, and whether there were confirmed or suspected COVID-19 cases in the county they live.

The second and third parts presented key outcomes of the survey: levels of vaccine hesitancy (attitude), general perceptions of vaccination, and perceptions of a potential COVID-19 vaccine. In the second part, general attitude and perceptions toward vaccination capture the current vaccination status and past experiences with vaccination of an individual, and serve as the baseline to investigate perceptions about a newly developed vaccine (the COVID-19 vaccine in this case). To measure general vaccine hesitancy, a question based on the definition by SAGE was used: “Have you ever hesitated, delayed, or refused about getting a vaccination for yourself due to reasons other than allergies and sickness?” (World Health Organization, 2014). The study also adopted six items from the Vaccine Confidence Index (VCI) survey tool and related studies to measure general vaccination perceptions, with four on confidence in the vaccine (importance, safety, effectiveness) and two on the degree of trust in sources of vaccination (health workers and governments) (de Figueiredo et al., 2020).

In the third part, intention to vaccinate with the COVID-19 vaccine was first assessed with the question “If a COVID-19 vaccine is successfully developed and approved for marketing in the future, would you accept vaccination?” with answers on a four-point Likert scale (“definitely yes,” “probably yes,” “probably no,” and “definitely no”). Then, to measure perceptions of the COVID-19 vaccine, confidence in the vaccine (importance, safety, and effectiveness) was assessed with same measures as for general vaccination perceptions. In addition, two items on vaccine complacency (perceived risk of infection and severity of COVID-19 disease) were assessed, which were incorporated in the “3Cs” model of vaccine hesitancy (World Health Organization, 2014; Dubé and MacDonald, 2016). The questions in the questionnaire were closed-ended and treated as categorical variables (see Supplementary Material). Items on self-reported health status and vaccination perceptions in general were assessed on a five-point Likert scale. Items on confidence in the safety and effectiveness of the COVID-19 vaccine were assessed on a six-point Likert scale, including “Unknown/not sure.”

### Statistical Analysis

To describe general vaccine hesitancy, the participants were classified into three groups. Those who had not delayed or refused, or had no doubts about vaccination were categorized as “no hesitancy.” Those who had hesitated or delayed but did not refuse vaccination were categorized as “hesitancy.” Those who had refused vaccination were categorized as “refusal.” The intention of the participants to accept a future COVID-19 vaccine were divided into three categories: “accepted” (definitely yes), “undecided” (probably yes or probably no), and “refusal” (definitely no). Univariate analyses were first performed by Chi-square tests to explore differences in demographics, general perceptions of vaccination, and perceptions of COVID-19 vaccine among the groups with different levels of general vaccine hesitancy. The Sankey diagram was used to show the distribution (flow) of intentions to accept future COVID-19 vaccine at different levels of general vaccine hesitancy. Logistic regressions were applied to examine the association of general vaccine confidence (independent variable), including safety, importance, and effectiveness, with vaccine confidence for a specific new COVID-10 vaccine (dependent variable), respectively.

To examine the impact of general vaccination attitudes and perceptions on the acceptance of a newly developed COVID-19 vaccine, generalized ordered logistic regression and path analysis models were used. The generalized order logistic regression has similar interpretation in explaining coefficients as multinomial logistic regression that was widely used in studies on vaccination acceptance. However, it allows for the inclusion of ordinal or hierarchy characteristic of vaccine attitude in the analysis and requires less stringent model specification (as in the partial proportional odds model) compared to the traditional ordered logit model (the proportional odds model) (Williams, 2016). Four sets of generalized ordered logistic regression were applied, all of which were adjusted for demographic characteristics and the situation of the pandemic. Model 1 included general vaccine hesitancy (attitudes), as the independent variable, to identify its association with acceptance of the COVID-19 vaccine. Models 2 and 3 included perceptions of vaccination in general and perceptions of COVID-19 vaccine, respectively, to examine whether these variables influence acceptance. In model 4, the variables in the above models were all included.

Based on the results of logistic regressions, the path analysis further examined inter-relationships (the direct and indirect effects) among the variables and tested the following hypotheses: (1) general perceptions (importance, safety, and effectiveness) of vaccination influence perceptions of COVID-19 vaccine; (2) general perceptions of vaccination influence acceptance of the newly-developed COVID-19 vaccine; (3) general perceptions of vaccination influence acceptance by directly influencing perceptions of the COVID-19 vaccine. To facilitate analysis, the items measured on the Likert scale, including health status and perceptions of vaccination, were merged into two categories for descriptive statistics and logistic regressions analysis. In the path analysis, measures of the independent and dependent variables were used in the Likert-scale form, and for measures of confidence in the safety and effectiveness of COVID-19 vaccine, the answer “don’t know” was merged with “neutral” to generate a five-point Likert scale for consistency. Odds ratios (ORs), 95% confidence interval (95% CI), and *p*-values were calculated. Statistical significance was established at an alpha (α) of *p* < 0.05. All data were analyzed using Stata 16.0 (Stata-Corp, College Station, TX, United States).

## Results

### Participant Characteristics and Vaccine Hesitancy Level

Table 1 presents the basic demographic characteristics of the participants and the situation of the pandemic by vaccine hesitancy level. Of the total 2,013 participants, about half (57.2%) were between the ages of 26 and 40, and 60.3% had an associate degree or a bachelor’s degree. Among the participants, 49% were male, 72.3% were married, and 85.1% were employed. The majority (48.6%) reported a total annual household income of between CNY 50,000 to CNY 150,000 (USD 7,246–21,739) in 2019. Approximately 67.9% reported having good/very good health, and 12.6% had chronic diseases. In terms of location, 83.5% lived in urban areas, and 65.1% lived in the eastern region. During the survey periods, 28.7% reported having COVID-19 cases in the county they lived.

**TABLE 1:** Participants’ characteristics and general vaccine hesitancy level, n (%).

Characteristics	Total sample	General vaccine hesitancy level			
		No hesitancy	Hesitancy	Refusers	*p*-value
Total	2013 (100)	1008 (50.1)	568 (28.2)	437 (21.7)	
**Age**					0.14
18∼25	332 (16.5)	164 (49.4)	106 (31.9)	62 (18.7)	
26∼30	434 (21.6)	199 (45.9)	127 (29.3)	108 (24.9)	
31∼40	717 (35.6)	377 (52.6)	181 (25.2)	159 (22.2)	
41∼50	360 (17.9)	181 (50.3)	110 (30.6)	69 (19.2)	
>51	170 (8.4)	87 (51.2)	44 (25.9)	39 (22.9)	
Male	987 (49.0)	530 (53.7)	264 (26.8)	193 (19.6)	0.005
**Education**					0.19
Middle school and below	111 (5.5)	58 (52.3)	20 (18.0)	33 (29.7)	
High school	585 (29.1)	294 (50.3)	165 (28.2)	126 (21.5)	
Associate or Bachelor	1214 (60.3)	608 (50.1)	350 (28.8)	256 (21.1)	
Master and above	103 (5.1)	48 (46.6)	33 (32.0)	22 (21.4)	
Married	1456 (72.3)	744 (51.1)	401 (27.5)	311 (21.4)	0.33
Employed	1714 (85.1)	864 (50.4)	475 (27.7)	375 (21.9)	0.49
**Total family income in 2019**					0.09
≤CNY 50,000	207 (10.3)	105 (50.7)	55 (26.6)	47 (22.7)	
CNY50,000–100,000	490 (24.3)	224 (45.7)	152 (31)	114 (23.3)	
CNY 100,000–150,000	489 (24.3)	232 (47.4)	139 (28.4)	118 (24.1)	
CNY 150,000–200,000	395 (19.6)	205 (51.9)	114 (28.9)	76 (19.2)	
≥CNY 200,000	432 (21.5)	242 (56.0)	108 (25.0)	82 (19.0)	
having a good/very good health	1366 (67.9)	734 (53.7)	381 (27.9)	251 (18.4)	<0.001
Having chronic disease	254 (12.6)	106 (41.7)	70 (27.6)	78 (30.7)	0.001
Urban	1680 (83.5)	850 (50.6)	487 (29.0)	343 (20.4)	0.005
**Location**					0.039
East	1311 (65.1)	658 (50.2)	350 (26.7)	303 (23.1)	
Central	409 (20.3)	213 (52.1)	116 (28.4)	80 (19.6)	
West	293 (14.6)	137 (46.8)	102 (34.8)	54 (18.4)	
Having COVID-19 cases in the county now	577 (28.7)	759 (52.9)	391 (27.2)	286 (19.9)	<0.001

The proportion of vaccine hesitancy to vaccination among the Chinese adult population is 49.9%, with 28.2% being hesitant and 21.7% refusing. The univariable analysis found significant differences in socio-demographics, including gender, health status, chronic disease status, region, and location among the participants with different levels of vaccine hesitancy.

### Perceptions of Vaccination in General and Perceptions of COVID-19 Vaccine and Intention to Accept the COVID-19 Vaccine

Table 2 shows the perceptions of vaccination in general and perceptions of COVID-19 vaccine and their association with general vaccine hesitancy. Of all the participants, 79.9% believed that vaccination was important for themselves, and 78.5% believed it was important for others. Regarding overall confidence in vaccines, 70.6% believed that vaccines were generally safe, and 75.8% believed that vaccines were effective. About 73.3% trusted health workers for information and suggestions on vaccination, and 79.6% trusted the government. Compared to general perceptions of vaccination, perceptions of the newly-developed COVID-19 vaccine were relatively favorable. Among all the participants, the proportions of individuals believing vaccination is important for themselves and others increased to 89.2 and 89.3%, respectively. Moreover, confidence in the COVID-19 vaccine also increased, with 84 and 84.8% believing in the safety and effectiveness of the new vaccine, respectively. Around 24.7% considered the risk of infection to be high, and 78.8% considered COVID-19 to be severe. More importantly, significant differences were observed in perceptions of vaccination in general, and perceptions of the new COVID-19 vaccine among those with different levels of vaccine hesitancy. Participants with no hesitation had the most positive perceptions compared to those with hesitation and refusal.

**TABLE 2:** Perceptions for vaccination in general and coronavirus disease 2019 (COVID-19) vaccine among participants with different general vaccine hesitancy levels.

Items	Total sample	General vaccine hesitancy level			
		No hesitancy	Hesitancy	Refusers	*p*-value ^c^
Total	2013 (100)	1008 (50.1^a^)	568 (28.2^a^)	437 (21.7^a^)	
**Believe the vaccination is important for oneself**					
For the vaccination in general^b^	1609 (79.9)	845 (83.8)	477 (84.0)	287 (65.7)	< 0.001
For the COVID-19 vaccination^b^	1796 (89.2)	924 (91.7)	520 (91.6)	352 (80.6)	< 0.001
Crude OR (95% CI)^d^	7.39 (5.48, 9.95)	9.38 (5.84, 15.07)	4.52 (2.41, 8.45)	5.67 (3.40, 9.45)	
Adjusted OR (95%CI)^e^	6.77 (4.90, 9.35)	8.92 (5.09, 15.65)	5.01 (2.42, 10.36)	6.42 (3.55, 11.6)	
**Believe the vaccination is important for others**					
For the vaccination in general^b^	1580 (78.5)	825 (81.8)	445 (78.4)	310 (70.9)	< 0.001
For the COVID-19 vaccination^b^	1797 (89.3)	914 (90.7)	521 (91.7)	362 (82.8)	< 0.001
Crude OR (95% CI)^d^	6.49 (4.83, 8.73)	6.02 (3.86, 9.39)	7.32 (3.90, 13.74)	5.92 (3.48, 10.06)	
Adjusted OR (95%CI)^e^	5.77 (4.21, 7.90)	5.47 (3.28, 9.13)	6.40 (3.10, 13.21)	9.98 (5.20, 19.16)	
**Believe the vaccine is safe**					
For the vaccination in general^b^	1422 (70.6)	772 (76.6)	388 (68.3)	262 (60.0)	< 0.001
For the COVID-19 vaccine^b^	1691 (84.0)	879 (87.2)	487 (85.7)	325 (74.4)	< 0.001
Crude OR (95% CI)^d^	4.78 (3.73, 6.14)	5.51 (3.74, 8.11)	3.68 (2.27, 5.97)	4.09 (2.60, 6.45)	
Adjusted OR (95%CI)^e^	4.49 (3.46, 5.82)	4.76 (3.14, 7.21)	3.52 (2.12, 5.84)	4.41 (2.65, 7.32)	
**Believe the vaccine is effective**					
For the vaccination in general^b^	1526 (75.8)	800 (79.4)	434 (76.4)	292 (66.8)	< 0.001
For the COVID-19 vaccine^b^	1707 (84.8)	881 (87.4)	492 (86.6)	334 (76.4)	< 0.001
Crude OR (95% CI)^d^	6.18 (4.78, 8.00)	6.89 (4.64, 10.22)	4.7 (2.84, 7.79)	5.76 (3.58, 9.26)	
Adjusted OR (95%CI)^e^	5.71 (4.36, 7.48)	6.23 (4.07, 9.55)	4.39 (2.49, 7.73)	6.11 (3.67, 10.18)	
Trust in health workers regarding vaccination information and suggestions^b^	1475 (73.3)	778 (77.2)	414 (72.9)	283 (64.8)	< 0.001
Trust in governments regarding vaccination information and suggestions^b^	1602 (79.6)	833 (82.6)	462 (81.3)	307 (70.3)	< 0.001
Perceive high infection risk of COVID-19^b^	498 (24.7)	243 (24.1)	134 (23.6)	121 (27.7)	0.26
Perceive high severity of COVID-19 disease^b^	1587 (78.8)	781 (77.5)	462 (81.3)	344 (78.7)	0.20

*^a^Row%.*

*^b^For each item, the number and proportion of the participants (in the total sample and different groups of vaccine hesitancy) who answered “very important/safe/effective/trustful” or “relatively important/safe/effective/trustful” are shown.*

*^c^Comparison of differences in items among respondents with different vaccine hesitancy levels by Chi-square tests.*

*^d^Binary logistic regression with perceptions for the vaccination in general as the independent variable and those for the COVID-19 vaccine as the dependent variable.*

*^e^Multiple logistic regression, adjusted for location, region, age group, gender, education, marital status, employment status, annual family income in 2019, health status, chronic disease status, and whether there are COVID-19 cases in a county.*

**FIGURE 1:**
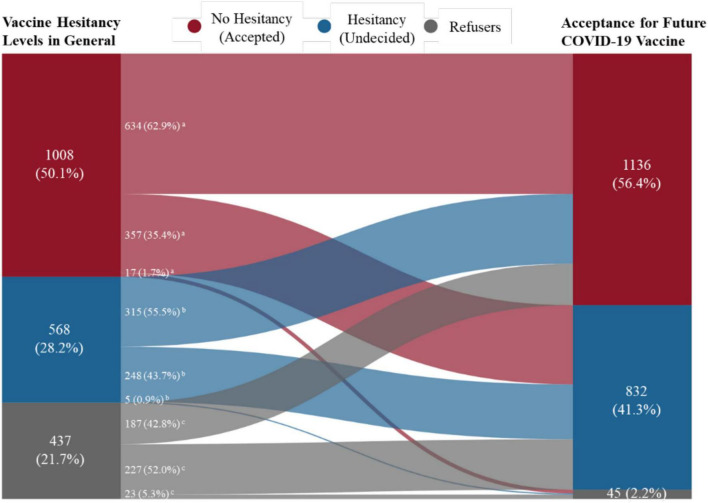
Acceptance for future coronavirus disease 2019 (COVID-19) vaccine among participants with different general vaccine hesitancy levels by with Sankey diagram. ^a^Number and proportion in “no hesitancy” general vaccine hesitancy group. ^b^Number and proportion in “hesitancy” general vaccine hesitancy group. ^c^Number and proportion in “refusal” general vaccine hesitancy group.

Regarding attitudes or acceptance of the future COVID-19 vaccine, the Sankey diagram (Figure 1) reveals that 56.4% would accept a future COVID-19 vaccine, while 41.3% stated they were undecided about the COVID-19 vaccination. Only 2.2% would refuse the COVID-19 vaccine. The acceptance levels of future COVDI-19 vaccine also varied significantly depending on the level of hesitation for the general vaccine. The acceptance rate for the COVID-19 vaccine was 62.9% for those with no hesitation about vaccination, 55.5% for those with hesitation, and 42.8% for those who refused. The rate of being undecided about the COVID-19 vaccine was also lowest for those with no hesitation toward general vaccination (35.4%), 43.7% for those with hesitation, and highest (52%) for those who refused (see Supplementary Table 1).

### Impact of General Vaccination Perceptions on Perceptions of the COVID-19 Vaccine

As shown in Table 2, binary and multiple logistic regressions identified the association between general vaccination perceptions (importance, safety, and effectiveness) and perceptions of the COVID-19 vaccine. The binary logistic regressions indicated that those who perceived vaccination to be important (for themselves and others), and vaccines to be safe and effective tended to have same positive perceptions of the new COVID-19 vaccine specifically. After adjusting for socioeconomic characteristics and the situation of the pandemic, the multiple logistic regressions were consistent with the effect of general vaccination perceptions on perceptions of the COVID-19 vaccine. For example, among all the participants, those who believed in the importance of vaccination in general were more likely to believe in the importance of the COVID-19 vaccination, both for themselves (aOR = 6.77; 95% CI = 4.9–9.35) and for others (aOR = 5.77; 95% CI = 4.21–7.9). Regarding confidence in vaccine, those who believed vaccines were safe were significantly more likely to believe that the COVID-19 vaccine was safe (aOR = 4.49; 95% CI = 3.46–5.82). In addition, those who believed the vaccine was generally effective were more likely to believe that the COVID-19 vaccine was effective (aOR = 5.71; 95% CI = 4.36–7.48). Among participants with different levels of general vaccine hesitancy, general vaccination perceptions had an impact on their perceptions of the COVID-19 vaccine.

### Impact of General Vaccination Attitudes and Perceptions on Acceptance of the COVID-19 Vaccine

In Table 3, the generalized ordered logistic regression models identify the association of general vaccination attitudes and perceptions with acceptance (attitudes) of the COVID-19 vaccine, all adjusted for socioeconomics characteristics and the situation of the pandemic. Model 1 includes only general vaccine hesitancy (attitudes) and shows that compared to those who refused, those with better attitudes (hesitancy or no hesitancy) toward vaccination in general were more like to show better acceptance of the future COVID-19 vaccine. Those with hesitancy in general vaccination were more likely to “accepted” (aOR = 4.7; 95% CI = 4.36–7.48) the future COVID-19 vaccine instead of “refusing or being undecided”, and they also had higher odds of being “accepted or undecided” than “refusals” (aOR = 1.62; 95% CI = 1.24–2.12) to take the vaccine. For those without hesitancy for vaccination in general, they had an equally higher odd (aOR = 2.22; 95% CI = 1.74–2.83) of being “accepted or undecided” than “refusals,” and being “accepted” compared to being “undecided or refused.” Model 2 controlled for vaccine hesitancy (attitudes) to show the impact of each measure on perceptions of general vaccination and on acceptance of the COVID-19 vaccine. In Model 2, those who believed that general vaccination was important to themselves were more likely to have an “accepted” attitude (aOR = 4.6; 95% CI = 2.32–9.14) instead of being “undecided or refusing” to accept the future COVID-19 vaccine. In Model 3, controlling for general vaccine hesitancy (attitudes), the association with perceptions of the COVID-19 vaccine was examined without the inclusion of general vaccination perceptions. The results suggested that belief in the importance of COVID-19 vaccine (both for oneself and others) and confidence in the future vaccine (safety and effectiveness) were both significant determinants of acceptance for the COVID-19 vaccine. With all the above variables included, model 4 found that a “no hesitancy” attitude toward general vaccination, compared to refusers, remained a significant determinant (aOR = 1.77; 95% CI = 1.36–2.31) of increase in the acceptance of the future COVID-19 vaccine. Perceptions of the COVID-19 vaccine remained the significant determinants of the acceptance of the COVID-19 vaccine. In terms of general perceptions of vaccination, after including different dimensions of perceptions of specific COVID-19 vaccines, those who considered vaccination to be important for themselves were also more likely to be “accepted” (aOR = 3.45; 95% CI = 1.66–7.2) than to be “undecided or refusing” to accept the future COVID-19 vaccine.

**TABLE 3:** Association of general vaccination attitudes and perceptions on the acceptance of the newly-developed COVID-19 vaccine by generalized order logistic regressions.

Variables	Model 1		Model 2		Model 3		Model 4	
	A vs. U, R	A, U vs. R	A vs. U, R	A, U vs. R	A vs. U, R	A, U vs. R	A vs. U, R	A, U vs. R
General vaccine hesitancy level								
Refusers	Ref		Ref		Ref		Ref	
Hesitancy	4.70** (1.81, 12.19)	1.62** (1.24, 2.12)	4.06** (1.55, 10.64)	1.54** (1.16, 2.03)	3.44** (1.36, 8.69)	1.29 (0.97, 1.72)	1.32 (0.99, 1.76)	1.32 (0.99, 1.76)
No Hesitancy	2.22** (1.74, 2.83)	2.22** (1.74, 2.83)	2.07** (1.61, 2.65)	2.07** (1.61, 2.65)	1.79** (1.38, 2.33)	1.79** (1.38, 2.33)	1.77** (1.36, 2.31)	1.77** (1.36, 2.31)
Believe the vaccination in general is important for oneself (yes vs. no)			4.60** (2.32, 9.14)	1.37 (0.99, 1.90)			3.45** (1.66, 7.2)	1.12 (0.77, 1.63)
Believe the vaccination in general is important for others (yes vs. no)			1.11 (0.84, 1.48)	1.11 (0.84, 1.48)			0.90 (0.66, 1.22)	0.90 (0.66, 1.22)
Believe the vaccination in general is safe (yes vs. no)			1.36* (1.05, 1.75)	1.36* (1.05, 1.75)			1.09 (0.83, 1.44)	1.09 (0.83, 1.44)
Believe the vaccination in general is effective (yes vs. no)			1.28 (0.97, 1.70)	1.28 (0.97, 1.70)			0.49* (0.25, 0.96)	1.06 (0.76, 1.47)
Believe the COVID-19 vaccination is important for oneself (yes vs. no)					3.82** (2.42, 6.04)	3.82** (2.42, 6.04)	3.61** (2.25, 5.77)	3.61** (2.25, 5.77)
Believe the COVID-19 vaccination is important for others (yes vs. no)					2.97** (1.96, 4.51)	2.97** (1.96, 4.51)	2.99** (1.94, 4.60)	2.99** (1.94, 4.60)
Believe the COVID-19 vaccine is safe (yes vs. no)					3.21** (2.26, 4.55)	3.21** (2.26, 4.55)	3.16** (2.22, 4.50)	3.16** (2.22, 4.50)
Believe the COVID-19 vaccine is effective (yes vs. no)					2.05** (1.44, 2.92)	2.05** (1.44, 2.92)	2.03** (1.42, 2.91)	2.03** (1.42, 2.91)
Trust in health workers regarding vaccination information and suggestions (yes vs. no)	1.50** (1.18, 1.89)	1.50** (1.18, 1.89)	1.17 (0.91, 1.50)	1.17 (0.91, 1.50)	1.16 (0.90, 1.50)	1.16 (0.90, 1.50)	1.11 (0.84, 1.45)	1.11 (0.84, 1.45)
Trust in governments regarding vaccination information and suggestions (yes vs. no)	3.03** (1.67, 5.50)	1.47** (1.14, 1.91)	1.06 (0.78, 1.44)	1.06 (0.78, 1.44)	1.02 (0.76, 1.37)	1.02 (0.76, 1.37)	0.98 (0.70, 1.39)	0.98 (0.70, 1.39)
Perceive high infection risk of COVID-19 (yes vs. no)	1.82** (1.43, 2.30)	1.82** (1.43, 2.30)	1.83** (1.44, 2.33)	1.83** (1.44, 2.33)	0.71 (0.31, 1.61)	1.63** (1.27, 2.09)	0.70 (0.31, 1.58)	1.63** (1.27, 2.09)
Perceive high severity of COVID-19 disease (yes vs. no)	2.95** (1.59, 5.49)	1.29* (1.02, 1.62)	2.72** (1.44, 5.12)	1.21 (0.96, 1.53)	1.08 (0.84, 1.39)	1.08 (0.84, 1.39)	1.09 (0.85, 1.40)	1.09 (0.85, 1.40)

All the models were adjusted for location, region, age group, gender, education, marital status, employment status, annual family income in 2019, health status, chronic disease status, and whether there are COVID-19 cases in a county. Adjusted odds ratio (OR) and 95% CI were presented. Significant level: **p < 0.01, *p < 0.05. R, Accepted (the COVID-19 vaccine); U, Undecided (to accept the COVID-19 vaccine); R, Refused (to accept the COVID-19 vaccine). A vs. U, R, U and R combined as the reference group, A as the comparison group; A, U vs. R, R as the reference group, A and U combined as the comparison group.

**FIGURE 2:**
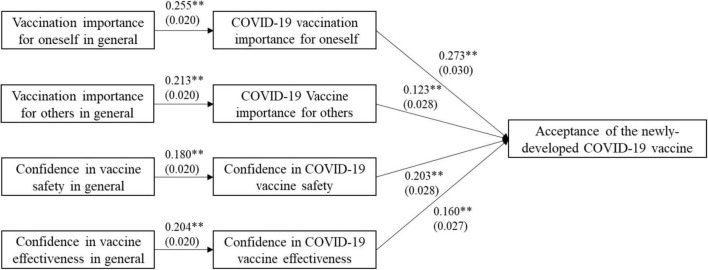
Path model on the impact of general vaccination perceptions on acceptance (attitude) of the COVID-19 vaccine. Coefficient and standard deviation were presented. Significant level: **p < 0.01.

Figure 2 presents the results of the preliminary path analysis examining the impact of general vaccination perceptions on acceptance (attitude) of the COVID-19 vaccine and the inter-relationship with perceptions of COVID-19 vaccine, controlling for general vaccine hesitancy, socioeconomic characteristics and the situation of the pandemic, perceptions of infection risk and the severity of COVID-19, and trust in health workers and governments. Having better perceptions of the COVID-19 vaccine, including the importance of COVID-19 vaccination for oneself and others, and confidence in the safety and effectiveness of COVID-19 vaccine, had a positive impact on the acceptance of future vaccination. First, hypothesis 1 was supported: better perceptions of vaccination in general positively influenced perceptions of the COVID-19 vaccine, which was similar to the results of the logistic regressions in Table 2. For example, general perceptions of the importance of vaccination were positively related to perceptions of the COVID-19 vaccine (β = 0.255, *SE* = 0.02, *p* < 0.001), and so was the impact of general confidence in the safety of vaccines on confidence in the safety of COVID-19 vaccine (β = 0.18, *SE* = 0.02, *p* < 0.001). Second, hypothesis 2 was partly supported: general perceptions of the importance of vaccination for oneself and confidence in vaccine safety have a positive overall effect on acceptance of the COVID-19 vaccine, but perceptions of vaccination importance for others and confidence in vaccine effectiveness did not have effects (see Table 4). Third, hypothesis 3 was supported: the indirect effect of general perceptions of vaccination on acceptance for COVID-19 vaccine was significant, while the direct effect was not, suggesting that general perceptions of vaccination affect acceptance by the mediation of perceptions of the COVID-19 vaccine rather than direct affect acceptance.

**TABLE 4:** Effect of general vaccination perceptions on acceptance (attitude) of the COVID-19 vaccine by path analysis.

Variable		Coefficient	95% CI	*p*-value
Vaccination importance for oneself in general	Total effect	0.114	0.066–0.161	< 0.001
	Direct effect	0.044	−0.003–0.092	0.068
	Indirect effect	0.070	0.051–0.088	< 0.001
Vaccination importance for others in general	Total effect	0.007	−0.030–0.045	0.701
	Direct effect	–0.019	−0.057–0.019	0.332
	Indirect effect	0.026	0.013–0.039	< 0.001
Confidence in vaccine safety in general	Total effect	0.058	0.018–0.098	0.005
	Direct effect	0.021	−0.017–0.059	0.280
	Indirect effect	0.037	0.024–0.049	< 0.001
Confidence in vaccine effectiveness in general	Total effect	0.015	−0.026–0.057	0.466
	Direct effect	–0.017	−0.058–0.024	0.416
	Indirect effect	0.033	0.020–0.045	< 0.001

## Discussion

This survey, conducted during a well-contained period of the COVID-19 pandemic, finds that the rate of vaccine hesitancy among the Chinese adult population toward general vaccination is 49.9%, and that socioeconomic characteristics and perceptions of general vaccination (e.g., vaccination importance, confidence in vaccine safety and effectiveness) vary significantly among the groups with different general vaccination attitudes. More importantly, this study indicates that psychological characteristics, especially general vaccination attitudes and perceptions, could influence the acceptance of the newly developed vaccine. First, general vaccination attitudes (vaccine hesitancy levels) are associated with perceptions of the new COVID-19 vaccine, and with acceptance of the future vaccination. Second, better general perceptions of vaccination, in terms of importance of vaccination and confidence in vaccine, have a positive impact on related perceptions of the COVID-19 vaccine among all the participants with different levels of general vaccine hesitancy. Finally, perceptions of vaccination importance to oneself and confidence in vaccine safety have a significantly positive total effect on the acceptance of future COVID-19 vaccine. While perceptions of vaccination importance for others and confidence in vaccine effectiveness may have an impact on related perceptions of the new vaccine, they did not have a significant effect on future acceptance of COVID-19 vaccination.

A few studies have assessed COVID-19 vaccination acceptance in China, especially during the severe period of the COVID-19 pandemic, with reported acceptance rates ranging from 82.3 to 91.3% (Lin Y. et al., 2020; Wang et al., 2020; Liu et al., 2021). Compared to findings during the severe pandemic, this study identified a relatively decline in acceptance rate, with 41.3% of the participants undecided about vaccination. This might be due to changes in the COVID-19 pandemic period over time in China and to the difference in question design for evaluating public vaccine hesitancy. Most previous studies have been conducted online with the general public. A comparative study between online and on-site surveys in China showed that 90% of online respondents accepted COVID-19 vaccination compared to the 82.1% in the on-site survey (Lyu et al., 2021). Recently, several studies have adopted a field design (Li et al., 2021; Wang et al., 2021b), focusing on the effect of geographic location, as well as on the elderly and population with non-communicable chronic diseases (NCDs). As for different regions, compared with the epidemic area (e.g., Hubei province), respondents in other regions had decreased intentions to get vaccinated (Li et al., 2021). In the preparation phase of the COVID-19 vaccination program in China, the willingness of the elderly to accept COVID-19 vaccine has been reported to be 79.08%, and no significant difference has been found between respondents with and without NCDs (Wang et al., 2021b). The participants in this study were mainly adults aged 18–50 years. The perceptions and attitudes of senior citizens toward vaccination in general and their impact on the COVID-19 vaccination may be one of the future research topics, and on-site surveys should be conducted wherever possible to obtain results from different regions.

Researchers investigating the hesitancy or acceptance of specific vaccines (e.g., influenza or HPV vaccines), such as the WHO SAGE group, have focused specifically on assessing attitudes, beliefs, or psychological perceptions of the population, including the importance of vaccination and confidence in vaccine safety or effectiveness, as these aspects are significantly associated with vaccine uptake and might be interfered by targeted campaigns or strategies (Velan et al., 2012; Wheelock et al., 2013; Barello et al., 2021; Murphy et al., 2021). Previous studies on influenza vaccines in the public have shown that vaccination experience is an important factor for accepting new influenza vaccines. Individuals who had been vaccinated against influenza in the past perceived higher levels of benefits from the vaccine and lower barriers to accessing new vaccines than those who had not been vaccinated (Shahrabani and Benzion, 2012). Furthermore, this study supports the view that psychological perceptions of general vaccination, which overall captures previous vaccination experiences and status of individuals, are also significantly associated with perceptions and acceptance of a newly developed vaccine against an emerging epidemic disease.

General vaccine hesitancy and perceptions are vital issues in vaccination, but only few studies have assessed the baseline of public attitudes and perceptions toward vaccination in general first before investigating the acceptance of a specific vaccine. Moreover, vaccine hesitancy should be interpreted in a particular context, especially in the context of specific population characteristics (Dubé and MacDonald, 2016; de Figueiredo et al., 2020). Previous studies have shown that negative information about vaccines could disrupt public confidence in vaccinations globally, as well as in China (Larson et al., 2011; Cao et al., 2018; Callaghan et al., 2021), and this study inferred the hesitancy level of general vaccination in China. In addition, this lack of knowledge about the distribution of vaccine hesitancy or different attitudes toward vaccination among the population studied would hinder the possibility of identifying key groups targeted for intervention in advance, while campaigns to promote vaccination perceptions and beliefs may need to be initiated routinely or before vaccination programs for a new vaccine begin (Velan et al., 2012; Mendel-Van Alstyne et al., 2018; Romer and Jamieson, 2020). This study indicates that positive attitudes rather than hesitancy toward general vaccination are associated with positive acceptance of new COVID-19 vaccine. Furthermore, the government and national vaccination authorities need to strengthen the knowledge and perceptions of general vaccination and eliminate hesitancy in order to prevent sudden epidemics of emerging infectious diseases and prepare for the uptake and acceptance of a new vaccine.

As with previous results for the H1N1 influenza vaccine and other newly developed vaccines, studies have provided useful information on urging effective strategies to address prevailing hesitations toward the new COVID-19 vaccine, which are largely due to uncertainty about vaccine safety, concerns about vaccine effectiveness, or negative attitude toward the need for vaccination (Zijtregtop et al., 2009; Eastwood et al., 2010; Raude et al., 2010; Brien et al., 2012; Valido et al., 2018; Lin C. et al., 2020; Chu and Liu, 2021; Robinson et al., 2021). Although experience of vaccination has been suggested as a determinant in vaccine acceptance, whether the general vaccination attitude and perceptions of an individual have an impact on their perceptions of a newly developed vaccine and the extent and mechanism of the impact remain an under-explored field (Marlow et al., 2007; Shahrabani and Benzion, 2012; Wheelock et al., 2013). This study suggests that beliefs in the importance for oneself and safety have a total effect on the acceptance of a new vaccine by indirectly affecting perceptions on vaccination importance for oneself and confidence in vaccine safety of COVID-19 vaccine. A study from the United States has shown that participants with higher perceived benefits of vaccine would show higher positive attitudes toward the COVID-19 vaccine and greater intention to vaccinate (Borah et al., 2021). A large-scale global retrospective analysis has revealed that confidence in the importance of vaccines has strongest association with vaccine uptake compared to other determinants considered (de Figueiredo et al., 2020). Vaccine safety is the primary concern for the public who questions vaccines (Larson et al., 2011; Chu and Liu, 2021), and a case of illegal vaccine sales related to vaccine safety in China has caused lack of confidence among vaccination recipients, and it would take a considerable time to eliminate the negative stigma associated with vaccine safety (Cao et al., 2018). The human papillomavirus (HPV) vaccine is effective in preventing cervical cancer and has been approved in recent years as a newly developed vaccine in China, and a survey reported that more positive perceptions of the importance and safety of the HPV vaccine were significantly correlated with intention to receive HPV vaccination (Liu et al., 2018). Previous studies have shown the associations of importance and safety of vaccine and acceptance and uptake of vaccine (Larson et al., 2011; Cao et al., 2018; Liu et al., 2018; de Figueiredo et al., 2020), and this study further explains that the mechanism by which perceptions of general vaccination importance and confidence in vaccine safety influence the acceptance of newly developed vaccine, which provides the evidence for focus in general vaccination cognition on raising public awareness of the importance of vaccines and ensuring the safety of vaccines. Perceptions of vaccination in general in different dimensions indirectly influence acceptance by mediation of perceptions of the COVID-19 vaccine rather than directly influence acceptance. This informs us that perceptions and acceptance of a newly developed vaccine does not only depend on perceptions of general vaccination and experiences of past vaccination. Therefore, we should take other special campaigns about benefits offered to people with new-developed vaccines, and strengthen the health literacy in preparedness policies for emerging infectious diseases (Levin-Zamir, 2020; Milošević Đorđević et al., 2021), as it is not enough to rely on the accumulation and experience of peacetime vaccination.

This study reports for the first time on the distribution and prevalence of general vaccination hesitancy in an adult population in China and used a variety of analysis methods to determine the impact of past and general vaccination attitudes and perceptions on the acceptance of a newly developed vaccine from a quantitative perspective. In addition, this survey was conducted at a time when a newly developed COVID-19 vaccine was not yet available to the public; thus the views of the population would not have been affected by vaccination campaigns or any health promotion. There are some limitations in this study. First, the use of an online survey may limit the representativeness of the results. A large sample size and stratified sampling were adapted to attempt to mitigate this limitation. Second, some self-reported answers may be biased because of factors such as information recall or social expectations. In particular, the question on future COVID-19 vaccination intention may not be certain to probe whether the true attitude of the participants is hesitation or refusal, as the participants may answer “being hesitant” but actually “being refusing” because of politeness or other reasons. Third, acceptance of the COVID-19 vaccine might differ from the actual level of acceptance of the vaccine. Further studies could focus on general vaccination attitudes, perceptions, and uptake of COVID-19 vaccination after the launch of a national vaccination campaign.

## Conclusion

This study assessed the general attitudes and perceptions of vaccination in a Chinese adult population, demonstrating a relatively high rate of vaccine hesitancy. Vaccination attitudes and perceptions in general are significantly associated with perceptions of the COVID-19 vaccine and future vaccination intention. In order to prepare for promoting vaccination with possible newly developed vaccines against future emerging diseases, routine health education and campaigns should be launched by related governments and vaccination authorities to improve public perceptions and cognition of vaccination in general, and to ensure optimal vaccination experience. Notably, raising public awareness of vaccination importance and ensuring confidence in vaccine safety will be two of the priorities. In addition, since general perceptions for vaccination could influence COVID-19 acceptance only indirectly as mediators, awareness-raising campaigns and mobilization specifically for newly developed vaccines would be important in the face of the emergence or resurgence of some specific infectious diseases, such as the current COVID-19.

## Data Availability Statement

The raw data supporting the conclusions of this article will be made available by the authors, without undue reservation.

## Ethics Statement

The study was approved by Peking University Institutional Review Board (IRB00001052-20011). The patients/participants provided their written informed consent to participate in this study.

## Author Contributions

RJ: conceptualization, data curation, formal analysis, investigation, methodology, visualization, writing (original draft), funding acquisition, and writing (review and editing). HF: conceptualization, funding acquisition, and supervision. HW: conceptualization, methodology, visualization, funding acquisition, supervision, validation, and writing (review and editing). JW: conceptualization, data curation, formal analysis, investigation, methodology, visualization, writing (original draft), supervision, validation, and writing (review and editing). All authors: final approval of the version to be published.

## Conflict of Interest

The authors declare that the research was conducted in the absence of any commercial or financial relationships that could be construed as a potential conflict of interest. The handling editor YB declared a shared affiliation with the authors JW, HF at the time of review.

## Publisher’s Note

All claims expressed in this article are solely those of the authors and do not necessarily represent those of their affiliated organizations, or those of the publisher, the editors and the reviewers. Any product that may be evaluated in this article, or claim that may be made by its manufacturer, is not guaranteed or endorsed by the publisher.
