# Supplementary material for: The Role of General Attitudes and Perceptions Towards Vaccination on the Newly-Developed Vaccine: Results From a Survey on COVID-19 Vaccine Acceptance in China
Source: Front Psychol. 2022 May 31;13:841189. doi: 10.3389/fpsyg.2022.841189 (PMC9194573; doi:10.3389/fpsyg.2022.841189)
Supplement: Supplementary file 1 [file Table_1.DOCX]

**Supplementary Material**

**S_Questionnaire**

China has reached the stage of regular epidemic prevention and control of the COVID-19 pandemic. COVID-19 vaccines are being developed, some of which are under phase Ⅲ clinical trials or emergency use. So far, no COVID-19 vaccine has been officially approved or available for the public in China. We would like to invite you to participate in the survey on the vaccination intention of the COVID-19 vaccine. By filling out the questionnaire, you agree to participate in the study. Your participation is completely voluntary. You have the right to withdraw from the study at any time. This questionnaire does not involve personal information and, in accordance with the regulations of the Statistics Law of the People's Republic of China, the answers will be kept strictly confidential.

1. **Basic Information**
2. Age: ①18~25; ②26~30; ③31~40; ④41~50; ⑤51~60; ⑥>60
3. Gender: ①Male; ②Female
4. Education level: ①Middle school and below; ②High school; ③Associate’s or bachelor’s degree; ④Master’s degree and above
5. Marital status: ①Married; ②Single; ③Divorced or widowed
6. Employment status: ①Employed; ②Unemployed
7. Annual household income in 2019 (RMB):①≤50,000; ②50,000~100,000;

③100,000~150,000; ④150,000~200,000; ⑤200,000~300,000; ⑥≥300,000

1. Health status: ①Very Good; ②Good; ③Fair; ④Poor; ⑤Very Poor
2. Do you have chronic disease(s)? ①Yes; ②No
3. Region: ①Urban ②Rural
4. Location (Province): ①Beijing; ②Tianjin; ③Hebei; ④Shanxi; ⑤Inner Mongolia;

⑥Liaoning; ⑦Jilin; ⑧Heilongjiang; ⑨Shanghai; ⑩Jiangsu; ⑪Zhejiang; ⑫Anhui; ⑬Fujian; ⑭Jiangxi; ⑮Shandong; ⑯Henan; ⑰Hubei; ⑱Hunan; ⑲Guangdong; ⑳Guangxi; ㉑Hainan; ㉒Chongqing; ㉓Sichuan; ㉔Guizhou; ㉕Yunnan; ㉖Tibet;

㉗Shaanxi; ㉘Gansu; ㉙Qinghai; ㉚Ningxia; Xinjiang

1. Have COVID-19 cases in the county you lived now: ①Yes; ②No
2. **Vaccine hesitancy** **level and perceptions for the vaccination in general**
3. Have you ever hesitated about getting a vaccination for yourself due to reasons other than allergies and sickness?

①Yes; ②No

1. Have you ever delayed about getting a vaccination for yourself due to reasons other than allergies and sickness?

①Yes; ②No

1. Have you ever refused about getting a vaccination for yourself due to reasons other than allergies and sickness?

①Yes; ②No

13. In general, how do you think about the importance of the vaccination for yourself?

①Very important; ②Relatively important; ③Fair; ④Relatively unimportant; ⑤Very unimportant

14. In general, how do you think about the importance of the vaccination for others?

①Very important; ②Relatively important; ③Fair; ④Relatively unimportant; ⑤Very unimportant

1. In general, how do you think of the safety of the vaccine?

①Very safe; ②Safe; ③Fair; ④Unsafe; ⑤Very unsafe

1. In general, how do you think of the effectiveness of the vaccine?

①Very effective; ②Effective; ③Fair; ④Ineffective; ⑤Very ineffective

17. Do you trust in health workers regarding vaccination information and suggestions?

①Very trustful; ②Relatively trustful; ③Fair; ④Relatively distrustful; ⑤Completely distrustful

18. Do you trust in governments regarding vaccination information and suggestions?

①Very trustful; ②Relatively trustful; ③Fair; ④Relatively distrustful; ⑤Completely distrustful

1. **Vaccine hesitancy level and perceptions for the COVID-19 vaccination**
2. If a COVID‐19 vaccine is successfully developed and approved for listing in the future, would you accept vaccination?

①Definitely yes; ②Probably yes; ③Probably no; ④Definitely no

13. How do you think about the importance of the COVID-19 vaccination for yourself?

①Very important; ②Relatively important; ③Fair; ④Relatively unimportant; ⑤Very unimportant

14. How do you think about the importance of the COVID-19 vaccination for others?

①Very important; ②Relatively important; ③Fair; ④Relatively unimportant; ⑤Very unimportant

1. How do you think of the safety of the COVID-19 vaccine?

①Very safe; ②Safe; ③Fair; ④Unsafe; ⑤Very unsafe; ⑥Unknown /not sure

1. How do you think of the effectiveness of the COVID-19 vaccine?

①Very effective; ②Effective; ③Fair; ④Ineffective; ⑤Very ineffective; ⑥Unknown /not sure

1. How do you think of your risk of COVID-19 infection?

①Very high; ②High; ③Fair; ④Low; ⑤Very low

1. How do you think of the severity of COVID-19 diseases?

①Very high; ②High; ③Fair; ④Low; ⑤Very low

Table S1. Acceptance for future COVID-19 vaccine among respondents with different general vaccine hesitancy levels, n (column %)

| Items | Total sample | General vaccine hesitancy level | | | |
| --- | --- | --- | --- | --- | --- |
|  |  | No hesitancy | Hesitancy | Refusers | *p*-value |
| **Total** | 2013 (100) | 1008 (50.1^a^) | 568 (28.2^a^) | 437 (21.7^a^) |  |
| Acceptance for future COVID-19 vaccine |  |  |  |  | **<0.001** |
| No Hesitancy | 1136 (56.4) | 634 (62.9) | 315 (55.5) | 187 (42.8) |  |
| Hesitancy | 832 (41.3) | 357 (35.4) | 248 (43.7) | 227 (52.0) |  |
| Refusal | 45 (2.2) | 17 (1.7) | 5 (0.9) | 23 (5.3) |  |

Note: ^a^ Row %.
